# RNA interference shapes stress responses in *Arabidopsis thaliana*

**DOI:** 10.3389/fpls.2025.1699198

**Published:** 2025-11-26

**Authors:** Elisa De Meo, Anna Baldisseri, Elena Loreti, Pierdomenico Perata

**Affiliations:** 1 PlantLab, Institute of Plant Sciences, Sant’Anna School of Advanced Studies, Pisa, Italy; 2 Institute of Agricultural Biology and Biotechnology, National Research Council, Pisa, Italy

**Keywords:** RNA interference, microRNA, Argonaute proteins, stress response, *Arabidopsis thaliana*

## Abstract

RNA interference (RNAi) is a crucial regulatory mechanism in plants, enabling dynamic responses to environmental stresses. Small RNAs (sRNAs), including miRNAs and siRNAs, guide stress-responsive gene silencing and can act beyond the cell of origin through systemic movement and uptake, enhancing plant adaptability. Central to these pathways are ARGONAUTE (AGO) proteins, whose stress-regulated expression and diverse roles at the molecular level contribute to finely tuned responses. This review integrates current progress in sRNAs systemic signaling, stress-responsive RNAi mechanisms, and AGO protein diversity into a coherent framework for understanding RNAi-driven stress adaptation. Emerging approaches such as high-resolution sRNA sequencing and single-cell transcriptomics are now enabling a deeper understanding of RNAi regulation with improved spatial and temporal resolution.

## 1 Introduction

### 1.1 RNA interference: overview and mechanistic background

The phenomenon known as RNA interference (RNAi) occurs in most living organisms but was initially observed in plants. Efforts to overexpress flavonoid biosynthesis genes to enhance floral coloration in transgenic petunias led to unexpected gene silencing effects (Napoli et al., 1990; van der Krol et al., 1990), which were only years later identified as due to RNAi. The first molecular mechanism explaining RNAi was obtained in the model organism *Caenorhabditis elegans* (Lee et al., 1993), where RNAi was found to be triggered by double-stranded RNA (dsRNA), with dsRNA injection causing a strong reduction in target mRNA levels (Fire et al., 1998). RNAi in *Caenorhabditis elegans* was also found to be capable of spreading from cell to cell (Fire et al., 1998).

The main actors in RNAi are 21–24 nucleotides (nt) long small (s)RNAs, that exist as micro (mi)RNAs or small-interfering (si)RNAs. miRNAs are 21–22 nt long, they originate from specific loci within the genome and are involved in post-transcriptional gene silencing (PTGS). siRNAs are 21/22/24 nt long, they are produced from long dsRNA of exogenous or endogenous origin (Vaucheret and Voinnet, 2024) and are associated with transcriptional gene silencing (TGS) and DNA methylation (Borges and Martienssen, 2015).

Double-stranded RNA precursors of endogenous miRNAs and siRNAs are processed into sRNAs by DICER-LIKE (DCL) endonucleases and subsequently loaded into ARGONAUTE (AGO) proteins (Borges and Martienssen, 2015). They form the RNA-induced silencing complex (RISC) and guide it to complementary nucleic acid targets, leading to their silencing (Voinnet, 2009).

The broad class of sRNAs comprises forms that differ in their biogenesis and function. As an example, it includes trans-acting siRNAs (ta-siRNAs), which are generated from non-coding transcripts in a phased manner and regulate gene expression similarly to miRNAs (Allen et al., 2005), as well as natural antisense siRNAs (nat-siRNAs), which derive from overlapping sense and antisense transcript pairs and typically function in stress responses (Borsani et al., 2005). tRNA-derived fragments (tRFs) are another class of sRNAs generated from tRNA processing, potentially regulating gene expression via the RNA silencing pathway. An analysis of the population of tRFs present in different *Arabidopsis* tissues and organs showed that some nuclear and chloroplast tRFs associate specifically with AGO1 (Cognat et al., 2017).

The growing list of sRNAs highlights the complexity and diversity of RNAi pathways in regulating plant development, genome stability, and responses to environmental cues.

### 1.2 RNA interference in plant stress biology

RNAi plays a central role in plant stress biology, which allows for rapid and reversible responses to environmental challenges through the regulation of gene expression at levels beyond transcriptional control (Betti et al., 2020). By dynamically modulating sRNA populations, plants adjust gene activity in response to stress type, intensity, and duration.

Both siRNAs and miRNAs fine-tune gene networks to enhance resilience under abiotic stresses, such as drought, salinity, temperature extremes, and nutrient limitation (Martín-Merchán et al., 2023). These molecules function at transcriptional and post-transcriptional levels, influencing mRNA stability, translation, and chromatin structure (Sunkar and Zhu, 2004). tRFs also function as regulators of stress responses and key components in plant defense mechanisms (Swain et al., 2025).

During biotic stress, both endogenous and pathogen-derived sRNAs play a role in defense. Plant miRNAs reprogram transcription factor networks in response to infection, while virus-derived siRNAs (viRNAs) can silence viral genes directly, being loaded into plant AGOs and acting as part of the plant antiviral mechanism (Ding and Voinnet, 2007). In fungal interactions, both host-and pathogen-derived sRNAs engage in an RNAi-mediated molecular exchange, reflecting an ongoing evolutionary interplay (Khraiwesh et al., 2012).

In this regulatory network, AGO proteins play the role of central mediators, allowing sRNAs to guide gene silencing in a stress-responsive manner. Through its association with specific miRNAs and siRNAs, AGOs help with direct precise transcript regulation, contributing to the plant’s ability to adapt to both abiotic and biotic challenges. Notably, even the expression and activity of several AGO proteins are modulated by stress conditions, suggesting that AGO function is dynamically integrated into the plant’s general stress response (Mallory and Vaucheret, 2010).

RNAi thus combines biotic and abiotic signals to coordinate stress-responsive gene expression regulation. Genetic studies in *Arabidopsis thaliana* and crops highlight its key adaptive functions and its potential for engineering stress-tolerant plants (Kaur et al., 2021).

## 2 Beyond the cell: systemic movement and exchange of sRNAs in stress response

### 2.1 sRNAs movement in plants

Regulation of plant growth and adaptation relies on complex molecular processes, which include both cell-autonomous mechanisms, in which signaling occurs within individual cells, and non-cell-autonomous mechanisms, in which signals such as sRNAs move from cell to cell (Pyott and Molnar, 2015).

For example, in *Arabidopsis* fully developed leaves, the transcription of miR160 is confined to the vasculature, but the mature miRNA also accumulates in vascular and epidermal tissues (Brosnan et al., 2019). The levels of its target gene (*ARF17*) are significantly higher in both the vasculature and epidermis of *hyl1* mutants, which have impaired miRNA processing, compared to wild-type plants (Brosnan et al., 2019). Also, miR164a, b, and c isoforms are distributed in both endodermis and cortex, despite transcriptional hints that appear confined to the root stele (Brosnan et al., 2019). These gradients are likely the result of the movement of either miRNA precursors (pri or pre-miRNA) or mature forms, since the associated AGO1 protein binding mature miRNAs is still restricted to single cell layers (Brosnan et al., 2019). Another case of a cell-to-cell movement is observed in the *Arabidopsis* shoot meristem, where miR394 controls stem cell identity by repressing its target, the F-Box Protein LCR. The mature form of miR394 is detectable over the three distal cell layers of the shoot meristem, expanding beyond its transcription site in the protoderm to maintain the stem cell pool and ensure proper shoot development (Knauer et al., 2013).

The plant cell wall prevents direct contact between neighboring cells and restricts the free movement of molecules; symplastic movement is facilitated by plasmodesmata, which are tiny channels connecting adjacent plant cells involved in cell-to-cell transport. They consist of cytoplasm strands, called cytoplasmic sleeves, which cross the cell wall, and may contain extensions of the endoplasmic reticulum (ER), known as desmotubules. Because the size of plasmodesmata can be modulated, they are thought to be crucial for regulating intercellular transport, controlling the size-exclusion limit (the maximum size of molecules allowed to pass) (Fuchs and Lohmann, 2020). As an example, upon viral infection, RNA viruses replicate their genome via dsRNA intermediates, relying upon movement proteins to infect new cells. Viral dsRNA also triggers a plant defense: it induces a mechanism that causes callose to build up at plasmodesmata, effectively sealing these channels to limit virus spread. To counteract this, viral movement proteins act as “effectors” at the infection front, reducing callose levels and increasing plasmodesmata permeability, thus facilitating viral movement. The suppression of plant defenses by movement proteins appears to be a common strategy among different plant viruses, and the fine-tuning of plasmodesmata is essential for intercellular movement and thereby plant defense (Huang et al., 2023).

Modulation of plasmodesmata density, specifically secondary plasmodesmata (channels formed in existing cell walls), regulates the cell-to-cell movement of sRNAs and other macromolecules in *Arabidopsis* aerial tissues (Jay et al., 2025). However, the movement of silencing signals through these channels is likely complex and not confined to simple passive diffusion, as much of the supporting literature remains indirect and correlative (Devers et al., 2023; Voinnet, 2025).

sRNAs can move both short distances, from cell to cell, and long distances, systemically throughout the plant. siRNAs have been found to travel long distances from shoots to roots, directing DNA methylation in recipient tissues (Molnar et al., 2010; Lewsey et al., 2016). Movement through plasmodesmata in the *Arabidopsis* root tip and shoot apical meristem seems to be restricted to certain miRNAs, suggesting the transport mechanism recognizes or preferentially moves only specific sRNAs between cells (Brosnan et al., 2019). Both miRNAs and siRNAs have been isolated from the vascular stream of several plant species, implying a systemic movement of silencing signals between different plant organs (Liu and Chen, 2018).

**Figure 1:**
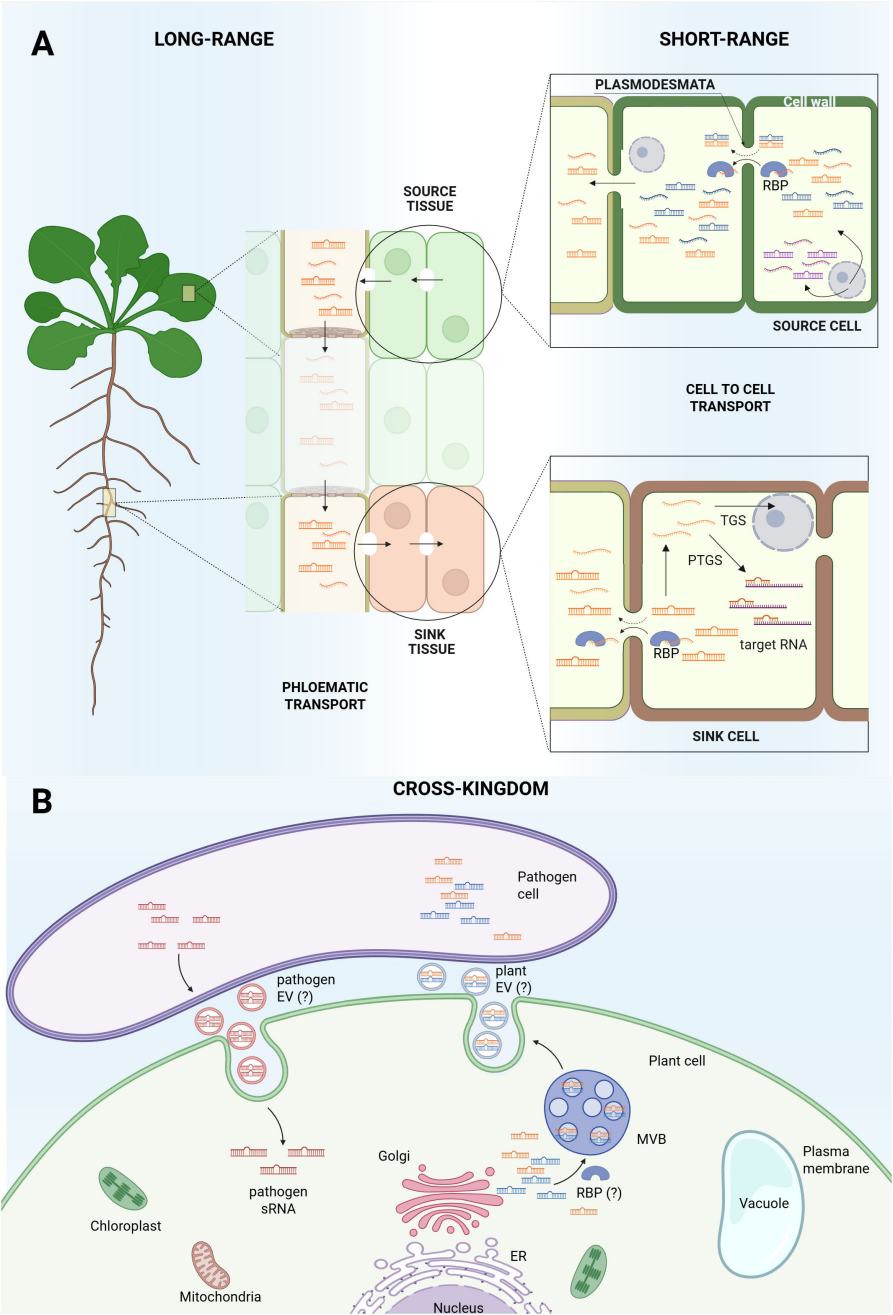
Small RNAs movement in *Arabidopsis thaliana*. **(A)** Biogenesis and movement of sRNAs within *Arabidopsis* plant. Some sRNAs are cell autonomous (represented in purple) while some sRNAs are produced by specific cells (source cell) and can move from one cell to another (sink cell), acting in a non-cell autonomous manner. sRNAs translocate from cell to cell through plasmodesmata, most probably as double strand sRNAs or as single strand sRNAs complexed with RNA-binding proteins (RBPs). Not all sRNAs seem to be systemically mobile (represented in blue), which supports the hypothesis that long-range movement through phloematic transport is restricted to specific RNA sequences (represented in orange) that act as mobile signals responsive to the plant’s physiological state. Once the sink cell is reached, sRNAs can interact with target RNA (PTGS) or enter the nucleus for TGS. **(B)** Cross-kingdom sRNAs transport. sRNAs present in the plant cytosol can be packaged into vesicles via the Golgi apparatus and secreted outside the cell, where they may be absorbed by plant pathogens. Pathogens can also generate sRNAs and deliver them into plant cells to modulate host gene expression. The question mark (“?”) indicates areas that remain unresolved, specifically, whether plants produce extracellular vesicles (EVs), whether other organisms can release EVs, and whether RBPs are involved in sorting sRNAs into EVs.

The role of RNA-binding proteins (RBPs) in this movement is diverse, as they can act as selective packagers for external transport or as dynamic consumers during intercellular spread. Endogenous, stress-induced miR399f, which regulates phosphate homeostasis, is suggested to traffic as primary protein-free duplex, while its precursors are confined, acting cell-autonomously (Chiang et al., 2023). This aligns with the AGO-mediated consumption model, which indicates AGO-unloaded sRNA duplexes as the mobile entities for cell-to-cell movement. According to this model, mobile sRNAs move across cell layers and are consumed by cell-autonomous AGO proteins, suggesting that the physical spread of the sRNAs does not equal their activity pattern (Devers et al., 2020). While sRNAs duplexes are thought to travel between plant cells as simple protein-free molecules, a family of RBPs recognizing and binding single-stranded sRNAs exists in plants (Yan et al., 2019). Their action seems to be both cell autonomous and non-cell autonomous, serving as possible mediators of sRNAs movement from cell to cell (Yan et al., 2019), as shown in **Figure 1**. Besides endogenous delivery or consuming, RBPs may play a distinct role in sRNAs export, acting in sorting and stabilizing plant-secreted sRNAs for transfer to fungal pathogens within extracellular vesicles (EVs) (He et al., 2021).

The presence of RNA species in the phloem sap supports the idea that plants use RNA as a long-distance signal, communicating the condition of source tissues to sink tissues at the whole-plant level (Thieme et al., 2015; Ham and Lucas, 2017).

### 2.2 Cell to cell sRNA signaling under stress conditions

Endogenously expressed miRNAs can act non-cell autonomously, meaning that they are produced in a cell but can move between cells, finally acting at a distance from the site of their synthesis. Although miR395 transcription is mostly confined to specific root or shoot areas, the mature miRNA form spreads to adjacent cells. During sulphate starvation, miR395 isoforms are transcribed only in some regions of the root tip: miR395c/e in the pericycle/inner stele and miR395a in the distal columella (Brosnan et al., 2019). The signal, however, spreads outwards to all surrounding cell layers, suggesting it moves from its source to neighboring cells as a response to sulphate starvation. Enhancing this miRNA movement likely helps increase the free sulphate pool during periods of starvation (Brosnan et al., 2019). After abscisic acid (ABA) stress exposure, vascular cells in *Arabidopsis* showed a more dynamic responsive pri-miRNA expression pattern than other cell types. This nature of miRNA expression, rapid and flexible, which differs from the more gradual shifts observed in broader transcriptome data, highlights its key role in the plant’s early stress response (Gao et al., 2025). Individual miRNAs may display distinct ABA response patterns across different cell types and time points, as miR164a and miR858a in vascular cells, which are candidates for involvement in cell-to-cell miRNA movement (Gao et al., 2025).

### 2.3 Systemic sRNA signaling under stress conditions

#### 2.3.1 Phloem sRNA composition

Given the capacity of sRNAs to regulate various transcription factor families, their levels are tightly controlled under stress within plant cells and organs. The observation that their amount in the phloem also changes in response to stress suggests they can be produced at the site of stress perception, then loaded into the phloem, and transported throughout the plant. The technical difficulties in providing examples of cell-to-cell movement of sRNAs originate from the challenge of observing and quantifying them, in distinct and specific cell types, within a complex and intact tissue. The challenges are mainly related to spatial resolution and to the nature of the mobile molecule itself. To demonstrate movement, it is essential to show that an sRNA detected in the recipient cell type originates exclusively from a spatially distinct source cell. Most conventional methods rely on bulk tissue analysis, which masks cell-to-cell variation. Moreover, it is unclear whether the mobile molecule is just naked sRNA, the sRNA duplex, a sRNA-AGO protein complex, or even packed within vesicles, making it more difficult to detect it (Ham and Lucas, 2017; Brosnan et al., 2019). Existing grafting systems have limited capacity to assess multiple RNA species at once and struggle to distinguish mobile sRNAs when identical sequences are present in both grafted tissues (Li et al., 2021). Moreover, studies often rely on non-native or modified reporter systems, which do not necessarily reflect the movement of an endogenously produced sRNA (Brioudes et al., 2021).

Analysis of *Arabidopsis* phloem under full nutrient growth conditions revealed 166 identifiable miRNA sequences (Bakirbas et al., 2023). Among these, the most abundant were isoforms of the miR166 family (a, b, c, d, e, f, g) and miR158a. Following these were isoforms of miR159a, b, and c, and miR165a and b (Bakirbas et al., 2023). Members of the miR165 and miR166 families have previously been reviewed as mobile over short distances (Zhan & Meyers, 2023) as in the *Arabidopsis* root tip they move from the endodermis to the outer stele, facilitating a process crucial for xylem cell specification (Brosnan et al., 2019). Also, isoforms of miR156, along with miR162, miR167, miR168, and miR169, have been identified in the phloem sap of *Arabidopsis*. The differing proportions of these miRNAs in the phloem suggest that their target-specific actions may be influenced by the physiological state of the plant. Additionally, phloem sRNA profiles are species-specific. For instance, under normal growth conditions, the sRNA patterns in *Brassica napus* phloem sap exhibit an enrichment of specific sRNAs compared to profiles in other tissues, including the inflorescence stem, leaves, and roots (Buhtz et al., 2010). miR162, miR167, miR168, miR169, and miR399 were more abundant in phloem than in the inflorescence stem, whereas others, such as miR158, miR396, and miR397, were enriched in the stem (Buhtz et al., 2010). Conversely, other miRNAs showed lower abundance in phloem sap compared to both leaves and roots (Buhtz et al., 2010). Furthermore, miR156 has been detected in the phloem cells of *Solanum tuberosum* plants, and grafting experiments involving an overexpressing genotype confirmed its shoot-to-root mobility, which plays a role in regulating tuberization under tuber-inductive conditions (Bhogale et al., 2014).

Under nutrient deficiency, plants need to adjust gene expression accordingly. Abiotic stresses, such as a nutrient-poor environment, can alter sRNAs production at a specific site, triggering a systemic response. A prime example of this is miR399: when *Arabidopsis* grows in phosphate-deprived conditions, miR399 is produced in the shoots and then moves to the roots (Bari et al., 2006; Pant et al., 2008). In iron-deprived conditions, miR830, miR857, miR5020b, miR5998a and b were identified in phloem exudate samples as being responsive to iron levels. They are detected only when plants are iron-deficient, suggesting they might become phloem-mobile specifically under these physiological conditions rather than being transported constitutively. Indeed, miR857 was particularly downregulated in whole shoots under iron deficiency but upregulated in the phloem sap (Bakirbas et al., 2023).

#### 2.3.2 sRNA mobility across plant organs

miRNAs found in the phloem may be dynamic signaling molecules capable of being transported through various plant organs. However, their presence in phloem sap is not directly correlated to its systemic long-distance transport throughout the entire plant (Buhtz et al., 2010).

miR399 is a known miRNA that translocates from shoots to roots via the phloem stream in response to phosphate starvation (Bari et al., 2006; Pant et al., 2008). To maintain phosphate homeostasis, *Arabidopsis* produces the miR399 duplex in the shoot under low-phosphate conditions, which then moves through the phloem to the roots. There, miR399 represses PHO2, an E2 ubiquitin-conjugating enzyme that targets the phosphate transporter PHT1 for degradation, enhancing phosphate uptake (Bari et al., 2006; Pant et al., 2008). This miR399–PHO2–PHT1 module coordinates phosphate demand in shoots with uptake in roots. Similarly, miR827 and miR2111a, also linked to ubiquitination processes, respond to phosphate starvation and exhibit shoot-to-root mobility, fine-tuning transporter activity to optimize nutrient uptake (Huen et al., 2017). miRNAs biologically active in post-transcriptional regulation originate from a miRNA duplex, consisting of the active strand (the guide strand) and its respective complementary strand. The complementary miRNA strand is known as the passenger strand or miRNA-star (miRNA*) and was generally considered a by-product of the miRNA biogenesis pathway, but it has been reported that it can also act as a regulatory factor (Liu et al., 2017). As miR399d, also miR399d* has been identified as a mobile signal candidate, demonstrating enrichment in the roots of grafts involving the *hen1* mutant (Huen et al., 2017). In *hen1* mutants, impaired methylation during biogenesis leads to a less stable miRNA duplex, resulting in significantly decreased miRNA levels (Park et al., 2002; Huen et al., 2017). Whether the miR399d* mobilization co-occurs with its companion strand requires further investigation, as it was also found to be enriched in *hen1* mutant shoots within grafts featuring a wild-type rootstock (Huen et al., 2017). This indicates that miR399d*, specifically, exhibits bidirectional mobility, moving between roots and shoots during periods of phosphate scarcity. The mechanisms and extent of mobility are not uniform across all miRNA species since miR399d and its star strand are mobile between shoots and roots, yet miR827 and miR2111a are mobile without their respective star strands (Huen et al., 2017). miR2111, miR169, miR827, and various other miRNA star strands within the phloem sap of another plant species, *Brassica napus*, are also significantly modulated by the plant’s phosphorus (or nitrogen) status (Pant et al., 2009).

Grafting experiments in *Arabidopsis* showed that miR395 accumulates under sulfur starvation and moves from shoots to roots through the phloem, repressing *APS4*, a key gene in sulfate assimilation (Buhtz et al., 2010). Translocation was also demonstrated between wild-type shoots and *hen1* mutant roots, indicating that the shoot-derived miR395 complements the mutant’s reduced miRNA levels. The fact that *hen1* mutants maintained their typical phenotype despite grafting with wild-type plants indicates that the translocation of some miRNAs is not sufficient to rescue all necessary miRNA functions (Buhtz et al., 2010).

miRNAs found in specific plant organs may not be detected in others, and the same applies to the phloem, which should act as a tissue required to translocate the miRNA. miR171a, similarly found in inflorescences but not in the phloem of *Arabidopsis* plants, showed no detectable translocation between grafted partners, supporting the idea that not all miRNAs are mobile (Buhtz et al., 2010). **Figure 1** schematically represents miRNA production and intercellular movement across *Arabidopsis* plants.

### 2.4 Cross-kingdom RNAi

sRNAs play key roles in inter-organism communication and their ability to move between cells, tissues, and even across species, enabling cross-kingdom interactions (Yan and Ham, 2022). Plant sRNAs can be found as exogenous molecules that, if taken up by other species, may integrate into regulatory pathways and influence gene expression in the recipient organism. They can be transferred between distinct plants, as in the case of hemi-parasitic plants and their host plant (Shahid et al., 2018), but they can also function as signaling molecules between plants and microorganisms, both symbionts and pathogens (Loreti and Perata, 2022). The plant-pathogen interaction and recognition trigger transcriptional reprogramming within the host, significantly involving sRNAs. This response activates both the plant’s internal protective mechanisms and active defense strategies, including signals directed at the pathogen. A clear example is observed in the interaction between the fungus *Botrytis cinerea* and *Arabidopsis*. At the initial stage of *Botrytis cinerea* infection, *Arabidopsis* produces plant sRNAs that are secreted via extracellular vesicles into *Botrytis cinerea* cells, with the aim of reducing fungal virulence by repressing fungal genes crucial for vesicle trafficking (Cai et al., 2018; Padilla-Padilla et al., 2024). In the interaction between *Arabidopsis* and *Botrytis cinerea*, the fungus not only receives sRNAs from the plant, but it actively secretes its own sRNAs inside extracellular vesicles. Once absorbed by the plant’s cells, these fungal sRNAs can suppress the genes responsible for plant immunity (Padilla-Padilla et al., 2024). Moreover, miR166 and miR159 are produced by *Arabidopsis* plants following infection by fungal pathogen *Verticillium dahliae*, which are then exported into the fungal hyphae to both silence specific fungal virulence genes and improve the plant’s ability to tolerate the disease (Zhang et al., 2016a). The parasitic plant *Cuscuta campestris* miRNAs function as trans-species regulators of host-gene expression when the plant parasitizes *Arabidopsis* as its haustoria accumulate high concentrations of miRNAs targeting host endogenous mRNA (Shahid et al., 2018). Predicted target sites for these miRNAs were also identified in homologous mRNAs from various other plant species, suggesting a broad regulatory effect. Cross-kingdom RNA interference has also evolved as an efficient mechanism for a plant and fungus to establish a symbiotic relationship, as in the symbiosis between *Arabidopsis* and the mutualistic fungus *Serendipita indica*, during which sRNAs from the fungus were shown to translocate into *Arabidopsis* root cells and load into the plant RNAi machinery (Nasfi et al., 2025).

The role of EVs in cross-kingdom sRNAs exchange has recently received growing attention. In *Arabidopsis*, EVs have been shown to carry a variety of sRNAs, including miRNAs, siRNAs and a class of 10–17 nucleotide “tiny RNAs”, indicating a selective loading mechanism for vesicle-mediated RNA transport (Baldrich et al., 2019). Some of these EVs accumulate at pathogen-infection sites and are taken up by pathogen cells, illustrating a role in cross-kingdom RNAi from plant to pathogen (Cai et al., 2018; Padilla-Padilla et al., 2024), as shown in **Figure 1**. More recent work also reveals heterogeneity among plant EV subpopulations in *Arabidopsis*, distinguished by markers such as TET8 and PEN1, pointing to potentially distinct biogenesis pathways and cargo-specific functions (Koch et al., 2025). However, the contribution of EVs in *Arabidopsis* remains poorly defined. Some evidence indicates that a large fraction of apoplastic sRNAs are located outside EVs, associated with RBPs rather than vesicles *per se*, raising the possibility of EV-independent sRNA secretion mechanisms (Zand Karimi et al., 2022; Koch et al., 2025). Together, these findings highlight that EV-mediated transport in *Arabidopsis* is a complex, multi-layered mechanism with significant implications for stress response and cross-kingdom communication, but that the roles of EVs and non-vesicular sRNAs still require further investigation.

The presence of RNA molecules coating the surface of *Arabidopsis* leaves and not protected from degradation by either EVs or RBPs was recently reported by Borniego et al. (2025). This leaf surface RNA differs from both apoplastic and cellular RNA in its size and composition, with tRNAs as the most abundant molecules in both the apoplast and on the leaf surface. Apoplastic tRNAs were generally processed into smaller tRFs, and tRNA-derived molecules are known to play a role in plant-microbe interactions. Specific miRNAs and siRNAs were also found outside the cell environment, suggesting they could be involved in shaping the leaf microbiome or in mediating immune responses (Borniego et al., 2025).

## 3 The molecular machinery: ARGONAUTE proteins in stress-responsive RNAi

### 3.1 AGO proteins in *Arabidopsis thaliana*

The name “Argonaute” originated from an early mutant identified in *Arabidopsis* that, because of missing AGO1, developed leaf shapes resembling the tentacles of the octopus *Argonauta argo* (Bohmert et al., 1998). Since then, AGO proteins have been recognized as highly conserved across bacteria, archaea, and eukaryotes, functioning as the core components of RNA-induced silencing complexes. They are central to RNA silencing, enabling guide RNA strand recognition, sequence-specific target cleavage, and recruitment of additional silencing factors (Wilson and Doudna, 2013).

Structurally, AGO proteins possess three conserved domains, which are PAZ, Middle (MID), and P-element induced wimpy (PIWI) domains. The N-terminal region, which includes the PAZ domain, is involved in binding the 3′ end of sRNAs and facilitating their separation from the target RNA. The C-terminal region harbors the MID and PIWI domains, where the 5′ end of the sRNA docks into a binding pocket formed between them. Importantly, the PIWI domain exhibits endonuclease activity analogous to RNase H, enabling AGO proteins to cleave target RNAs in a sequence-specific manner (Parker, 2010).

In *Arabidopsis*, the AGO protein family comprises ten members, which are phylogenetically grouped into three major clades: AGO1/5/10, AGO2/3/7, and AGO4/6/8/9 (Mallory and Vaucheret, 2010). Although AGO8 is often classified as a pseudogene due to a premature stop codon that probably disrupts its function, some reports suggest that it may still be transcribed under specific conditions (Vaucheret, 2008). Each AGO protein exhibits distinct binding preferences for the 5' nucleotide of associated sRNAs, a specificity primarily mediated by a loop in the MID domain that differentiates between 5'-U, -A, and -C. For instance, AGO1/10 favor 5'-U, AGO4/6/9 and AGO2/3/7 prefer 5'-A, while AGO5 predominantly binds sRNAs with 5'-C (Mi et al., 2008; Montgomery et al., 2008; Takeda et al., 2008; Zhang et al., 2016b).

Besides the 5′ nucleotide bias, other factors also determine AGO loading specificity, such as the duplex structure of the sRNA and its length. AGO1/10 and AGO2/7 generally associate with 21–22 nt sRNAs, including miRNAs and siRNAs, while AGO4/6/9 preferentially bind 24 nt sRNAs (Mi et al., 2008; Havecker et al., 2010; Wang et al., 2011; Zhu et al., 2011; Annacondia & Martinez, 2021). AGO3 and AGO5 exhibit broader loading capabilities, binding both 21 and 24 nt sRNAs (Zhang et al., 2016b; Marchais et al., 2019; Jullien et al., 2020). The molecular mechanisms underlying this length-based discrimination remain to be fully elucidated.

### 3.2 Stress-regulated subcellular localization of AGO proteins

AGO proteins function in both PTGS and TGS, and their subcellular localization, whether in the cytoplasm, nucleus, or membrane-associated compartments, is critical for defining their functional roles. For instance, AGO1 predominantly resides in the cytoplasm, where it mediates miRNA-guided translational repression and mRNA cleavage (Baumberger and Baulcombe, 2005), while AGO4 localizes to the nucleus, where it plays a central role in the RNA-directed DNA methylation (RdDM) pathway (Zilberman et al., 2003).

Under stress conditions, AGO localization becomes highly dynamic, adapting to changing environmental cues. Under normal circumstances, AGO1 is enriched at the rough endoplasmic reticulum (ER), where it facilitates miRNA-mediated regulation. However, heat stress triggers AGO1 phase separation into cytoplasmic condensates that co-localize with stress granules (SGs) and siRNA bodies (Blagojevic et al., 2024). This stress-induced relocalization is driven by a prion-like domain in the N-terminal polyglutamine-rich region and appears to protect AGO1 function without impairing sRNA loading or cleavage activity (Blagojevic et al., 2024). AGO2, in turn, shows pronounced changes in localization during bacterial infection. Upon pathogen challenge, AGO2 is strongly induced and partners with specific miRNAs such as miR393* to enhance immunity. This regulatory axis targets genes like MEMB12, a SNARE protein, promoting defense protein secretion (Zhang et al., 2011). Additionally, hormonal signals modulate AGO subcellular distribution. Abscisic acid (ABA) increases AGO1 expression and influences its nuclear-cytoplasmic balance, while brassinosteroids (BRs) regulate its association with the ER. BR-deficient mutants show higher ER-localized AGO1, highlighting hormonal control over AGO dynamics (Zhao et al., 2023).

### 3.3 Biological roles of AGO proteins in stress conditions

**Figure 2:**
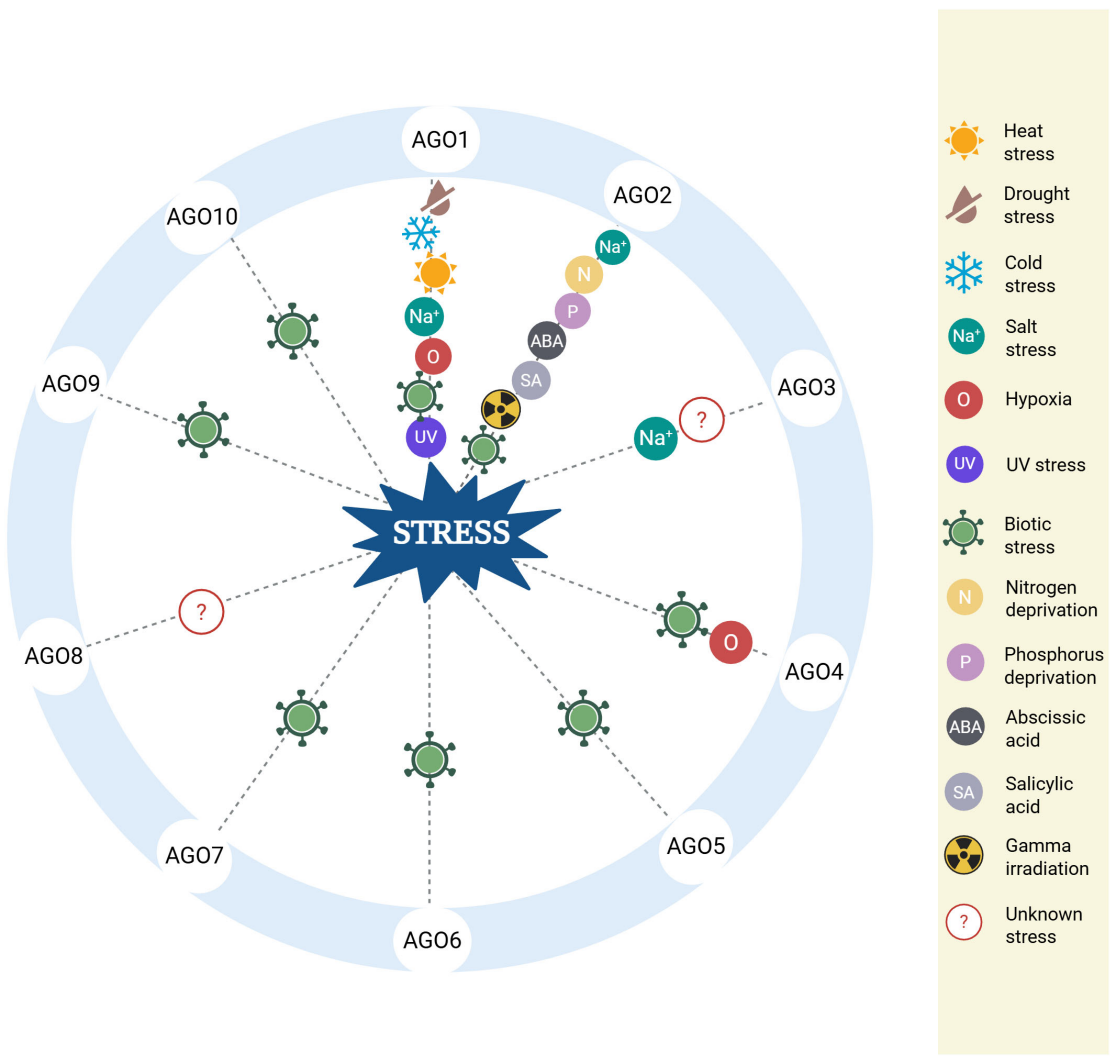
Biological roles of AGO proteins under various stress conditions. The diagram summarizes the role of specific AGO proteins in diverse stress responses in *Arabidopsis*. Each AGO is linked to one or more types of stress, including heat, drought, cold, salinity, hypoxia, UV exposure, nutrient deprivation, pathogen infection, hormone exposure, and gamma irradiation. The depicted associations are supported by experimental findings discussed in the main text. Full reference details for each stress-related function are provided in the corresponding sections of this review.

AGO protein expression in *Arabidopsis* is spatially and temporally regulated, with some members expressed constitutively across tissues and others activated only in specific cell types or in response to environmental stimuli (Martín-Merchán et al., 2023). Under stress, differential AGO expression supports precise regulatory responses that facilitate plant adaptation to biotic and abiotic challenges, as shown in **Figure 2**.

Under abiotic stress conditions, increased levels of AGO1 and miR168a during drought suggest a role in water deficit stress tolerance (Li et al., 2012). AGO1 also contributes to salt stress resilience. Under salinity stress conditions, the expressions of *MIR161* and *MIR173* are negatively regulated, as AGO1 cotranscriptionally controls the expression of these genes in the nucleus. More in detail, AGO1 interacts with chromatin at *MIR161* and *MIR173* loci and causes the disassembly of the transcriptional complex, releasing short and unpolyadenylated transcripts (Dolata et al., 2016). AGO2 contributes to salt stress tolerance by interacting with the RNA-binding protein MUG13.4, which is essential for its function in these conditions (Wang et al., 2011). AGO2 is also upregulated under nitrogen or phosphorus deprivation (Zhang et al., 2016b), and in response to ABA, salicylic acid, gamma-irradiation, and bleomycin treatment (Wei et al., 2012; Alazem et al., 2019). Together with AGO1 and AGO2, AGO3 also plays a critical role in salt stress response by associating mainly with 24 and activating the RNA-directed DNA methylation (RdDM) pathway (Zhang et al., 2016b). At the same time, other findings suggest that AGO3 may associate with both 24- and 21-nucleotide sRNAs and contribute to PTGS; therefore, its precise role within the RNAi pathway remains unresolved (Jullien et al., 2020b).

AGO1 is involved in responses to both cold and heat stress, operating through temperature-sensitive signaling pathways (Li et al., 2012), and contributes to UV damage repair by interacting with UV-induced sRNAs (uviRNAs) and interacting with DNA damage binding protein 2 (DDB2) (Schalk et al., 2017).

Both AGO1 and AGO4 have also been implicated in hypoxia responses (Loreti et al., 2020). More in detail, in *Arabidopsis*, hypoxia resulting from submergence causes extensive transcriptional reprogramming. While the impact of low oxygen on gene expression is well characterized, the role of RNAi in hypoxia responses has only recently begun to emerge. Notably, mutants deficient in AGO1 (e.g., *ago1-27*) show enhanced sensitivity to submergence stress, and transcriptome analyses have identified a set of hypoxia-responsive genes whose regulation is dependent on AGO1 function. Additionally, analysis of mutants led to a convergence between the AGO1-mediated post-transcriptional pathway and the AGO4-mediated RNA-directed DNA methylation (RdDM) pathway. Interestingly, methylation patterns are altered not only in *ago4–1* mutants but also in lines overexpressing a stable version of the oxygen sensor RAP2.12, suggesting an interplay between oxygen sensing and epigenetic regulation via RNAi (Loreti et al., 2020).

In the context of biotic stress, AGO1 plays a central role in plant immunity. During *Botrytis cinerea* infection, fungal sRNAs are loaded into AGO1 to suppress host defense genes. Notably, *ago1* mutants exhibit reduced susceptibility to infection, suggesting that *Botrytis cinerea* hijacks the host RNAi machinery (Weiberg et al., 2013). Additional AGO members, including AGO1, AGO2, AGO7, and AGO9, are implicated in defense against *Phytophthora* spp. and *Sclerotinia sclerotiorum*, where they modulate defense gene expression via PTGS (Cao et al., 2016; Guo et al., 2018). AGO1, AGO2, and AGO7 also promote defense against viral and bacterial pathogens (Garcia-Ruiz et al., 2015; Dunker et al., 2020). Recent studies have shown that enhanced plant resistance to bacterial infection is linked to the stability of AGO2. Specifically, a reduction in the expression of protein arginine methyltransferase 5 (PRMT5) leads to lower levels of AGO2 arginine methylation. This post-translational change prevents AGO2 degradation, resulting in its stabilization and accumulation, along with its associated sRNAs, thereby boosting the plant’s immune response (Hu et al., 2019).

AGO proteins are also central to antiviral immunity. AGO1, AGO2, AGO4, AGO5, AGO7, and AGO10 have all been implicated in antiviral defenses in *Arabidopsis* (Carbonell and Carrington, 2015). AGO1, for instance, is notably upregulated upon viral infection, where it binds virus-derived small interfering RNAs (vsiRNAs) and targets viral RNA for degradation. Its overexpression improves resistance to both turnip crinkle virus (TCV) and brome mosaic virus (BMV) (Zhang et al., 2012; Manacorda et al., 2025). During infection by turnip mosaic virus (TuMV), viRNAs guide AGO2 to cleave viral transcripts, underscoring its antiviral role (Garcia-Ruiz et al., 2015). AGO4 has been linked to resistance against *Plantago asiatica* mosaic virus, further demonstrating the multifaceted antiviral functions of the AGO family (Brosseau et al., 2016).

As summarized in **Figure 2**, AGO proteins combine sRNA pathways with diverse stress responses in *Arabidopsis*, allowing flexible and multilayered gene expression regulation. Their stress-inducible expression and specialized functions under stress conditions highlight their importance as critical molecular tools for survival and adaptation.

## 4 Discussion

The complex regulation of gene expression by RNAi is necessary to perceive, respond to, and survive many environmental stresses, allowing plants to fine-tune gene silencing with spatial and temporal precision. The ability of sRNAs to move between cells and over long distances reveals a complex level of systemic communication that plants use to coordinate stress responses across tissues (Melnyk et al., 2011). However, the exact molecular pathways they follow, and the barriers regulating their selective movement, are still largely unknown (Shine et al., 2022; Yan and Ham, 2022).

AGO proteins act as the effectors of RNAi, connecting sRNA recognition with target gene silencing. Stress-induced dynamic shifts in AGO activity and localization support a refined functional plasticity, proving that plants have evolved RNAi beyond a static silencing tool into a highly flexible network that fluently integrates environmental signals with internal regulatory pathways. Understanding how this functional plasticity takes place requires looking more closely at the diversity of AGO proteins and their often-overlapping roles.

A key aspect of limited understanding is the redundancy and specificity of AGO proteins. AGO3 and AGO8 functions are still unknown, and their mutants show no obvious phenotypes. The distinct contribution of AGO3 and its potential role under stress conditions are still not well defined, and finding out if AGO3 works uniquely or overlaps with other AGOs could uncover new layers of RNAi regulation (Martín-Merchán et al., 2023). AGO8 represents another element of complexity. Although generally considered a pseudogene due to a premature stop codon that likely inactivates it, some reports suggest AGO8 could still be transcribed under certain conditions (Vaucheret, 2008). This opens intriguing possibilities regarding AGO3 and AGO8 potential regulatory or decoy-like roles, especially in stress scenarios where non-canonical AGO activity might emerge.

Beyond specific RNAi components, most of our present knowledge comes from controlled experimental systems involving single, well-defined stressors under simplified laboratory settings, which do not resemble the complexity of natural environmental stresses plants encounter. The crosstalk between simultaneous abiotic and biotic stressors on RNAi pathways is still largely unclear, delaying the translation of molecular knowledge into practical strategies for crop improvement (Hirayama and Shinozaki, 2010). Overcoming these gaps will require new approaches that combine high-resolution, single-cell technologies with *in vivo* models of complex stress scenarios.

Systemic acquired resistance (SAR) is another frontier where the involvement of RNAi is still highly speculative. Even though sRNAs are implicated in SAR-related signaling, clear mechanistic links between RNAi machinery and the coordination of SAR responses are yet to be defined (Yan and Ham, 2022). Clarifying this aspect could help understand how local RNAi responses scale into whole-plant immunity.

Recent breakthroughs in high-throughput and precision technologies provide promising prospects to fill these gaps. The advent of single-cell transcriptomics provides a new opportunity to dissect RNAi activity at the cellular resolution, revealing spatial and temporal gene regulation patterns that are masked in bulk analyses (Schmitz et al., 2022). Also, sRNA sequencing has become increasingly sophisticated, enabling the identification of low-abundance and stress-responsive sRNAs under diverse developmental and environmental conditions (Li et al., 2023). These innovations will help understand how sRNA movement and AGO specialization participate in systemic signaling and stress memory, finally allowing the design of precise molecular tools.

In an era in which climate change increases plant stress challenges worldwide, analyzing the nuanced roles of sRNAs and AGO proteins is fundamental for developing resilient crops and guaranteeing global food security.
